# Testing the efficacy of the CaregiverTLC program to positively impact the caregiver’s physical and mental health

**DOI:** 10.1093/geroni/igaf122.2658

**Published:** 2025-12-31

**Authors:** Julian Montoro-Rodriguez, Charlie Reeve, Dolores Gallagher-Thompson, Ann Bilbrey, Jennifer Ramsey, Bruno Kajiyama

**Affiliations:** The University of North Carolina at Charlotte, Charlotte, North Carolina, United States; The University of North Carolina at Charlotte, Charlotte, North Carolina, United States; Stanford University, Stanford, California, United States; Optimal Aging Center, Sunnyvale, California, United States; York Technical College, Rock Hill, South Carolina, United States; Photozig, Inc., Moffett Field, California, United States

## Abstract

Providing care for a loved-one with a chronic health condition or significant neurocognitive disorders, such as dementia, is a demanding job. The demands of this job can often have an impact on these caregivers’ own physical and mental health. In this study, we examine the short-term efficacy of the Caregiver TLC psychoeducational program to attenuate this negative impact on caregivers’ self-reported general, physical and mental health as assessed by the SF-12 quality of life health survey. The Caregiver TLC is a skill-building program designed to improve caregiver internal and external coping mechanisms, delivered online to a small group of caregivers over six-weekly two-hour sessions (Montoro-Rodriguez et al., 2024; 2025). Using pre-post data from a randomized controlled trial (N = 78), we conducted a two-way repeated measures ANOVA to test for differences in change in scores over time on each of the three measures of health from the SF-12. Results indicated small to medium effects, with caregivers in the intervention group reporting higher levels of improvement in scores for the 3 sub-scales of the SF-12: overall health, physical health and mental health - compared to those in the control condition. These results suggest that although the Caregiver TLC programming is targeting caregiver coping mechanisms (e.g., building resilience, and increasing emotional support), caregivers gain additional benefits on more distal self-reported health outcomes. Our findings are consistent with previous empirical studies and systematic reviews of the effectiveness of psychoeducational interventions for caregivers that report significant improvement in a variety of health outcomes.

